# An Experimental Study on the Hot Alkali Extraction of Xylan-Based Hemicelluloses from Wheat Straw and Corn Stalks and Optimization Methods

**DOI:** 10.3390/polym14091662

**Published:** 2022-04-20

**Authors:** Adrian Cătălin Puițel, Gabriel Dan Suditu, Maricel Danu, Gabriela-Liliana Ailiesei, Mircea Teodor Nechita

**Affiliations:** 1 Faculty of Chemical Engineering and Environmental Protection “Cristofor Simionescu”, “Gheorghe Asachi” Technical University of Iasi, No. 73, 700050 Iaşi, Romania; puitelac@tuiasi.ro (A.C.P.); suditu_g@yahoo.com (G.D.S.); maricel.danu@academic.tuiasi.ro (M.D.); 2 “Petru Poni” Institute of Macromolecular Chemistry, 41A Grigore Ghica Voda Alley, 700487 Iași, Romania; gdarvaru@icmpp.ro

**Keywords:** hot alkali extraction (HAE), hemicelluloses, xylan, wheat straw, corn stalks, response surface method (RSM)

## Abstract

In this paper, we describe an experimental study on the hot alkali extraction of hemicelluloses from wheat straw and corn stalks, two of the most common lignocellulosic biomass constituents in Romania. The chemical compositions of the raw materials were determined analytically, and the relevant chemical components were cellulose, hemicelluloses, lignin, and ash. Using the response surface methodology, the optimum values of the hot alkaline extraction parameters, i.e., time, temperature, and NaOH concentration, were identified and experimentally validated. The physicochemical characterization of the isolated hemicelluloses was performed using HPLC, FTIR, TG, DTG, and 1H-NMR spectroscopy. The main hemicellulose components identified experimentally were xylan, arabinan, and glucan. The study emphasizes that both corn stalks and wheat straw are suitable as raw materials for hemicellulose extraction, highlighting the advantages of alkaline pretreatments and showing that optimization methods can further improve the process efficiency.

## 1. Introduction

It took a half of century for the lignocellulosic biomass (LCB) to be acknowledged as a consistent alternative to classic fossil fuels, i.e., petroleum, natural gas, and coal. Today, a brand new LCB-based industry is on the rise aiming to produce bioenergy [1,2,3,4], biofuels [5,6,7,8,9], biomaterials [10,11,12], and various biochemicals [13,14,15,16,17].

The common constituents of LCB are forest residues and agricultural wastes (including dedicated energy crops, algae biomass, grasses, organic municipal solid waste, and some industrial wastes (wood, paper, pulp, food)) [5,6,13,18]. From a chemical point of view, LCB is a complex material in which three biopolymers, i.e., cellulose, hemicelluloses, and lignin, are mixed and interlinked in various proportions, making up about 90% of the dry matter [2,8,18,19,20].

The undeniable advantages of LCB as a suitable and competitive raw material are that it is highly renewable, carbon-neutral, and reasonably inexpensive [4,15,16,20]. However, LSB has some major drawbacks, including: (i) its seasonal variation and spatial distribution (spatiotemporal availability) [1,13,21,22] which cause issues with collection, handling, and storage [13,23]; (ii) the complexity of LCB constituents and the variable chemical compositions, which requires specific, costly, and energy intensive pretreatments [14,24,25,26]; (iii) a lack of cost-effective and commercially developed technologies [18]. Currently, technical enterprises are making substantial efforts to overcome these limitations [27,28,29], with supportive legislation complementing the technological efforts [30,31].

Such a variety of LCB sources with such an assortment of raw constituents, coupled with the natural rigidity and recalcitrance of the lignocellulosic matrix have unavoidably led to the development of a multitude of pretreatment processes [32,33]. The main factors responsible for mechanical, physicochemical, and biochemical recalcitrance of LCB are the structural heterogeneity, cellulose crystallinity, the degree of lignification, and the complexity of cell-wall ingredients [32,33]. Except in the direct combustion of LCB, LCB pretreatments are compulsory and play essential roles in the technological pathways to biogas, bioethanol, biochemicals, biomaterials, and bioenergy [4,24].

Corn stalks and wheat straw are a category of LCB abundantly available in the European Union and especially, in Romania, which is the largest producer of corn stalks (23.3% of the EU’s total production) and in third place as a producer of wheat straw (12.1% of the EU’s total production) after France (22.9%) and Germany (12.3%) [7]. Most of the large-scale farms in Romania cultivate both wheat and corn as complementary crops, following a wheat-maize rotation system [34,35,36]. The harvesting period for wheat goes from the middle of June to the middle of August, while the harvesting period for corn goes from the beginning of September to late November. Consequently, “fresh” raw materials represented by wheat straw (WS) and corn stalks (CS) are available for nearly half of the year. Furthermore, WS and CS are both fairly easy and cheap to gather, transport, and store, as well as relatively stable to natural decay caused by the action of natural enzymes, yeasts, bacteria, and fungi as compared with other LCB constituents. In other words, when stored in proper conditions, they remain relatively stable in terms of chemical composition for at least a few months, enough to support hemicellulose production until the next harvesting season and to close the cycle. Therefore, a technological approach based on both WS and CS might be sustainable at least in regard to these raw materials.

The main technological challenge is to find an adequate pretreatment to ensure acceptable production yields from both WS and CS. Specific pretreatments for CS [25,37,38] and WS [26,39,40,41,42,43] have been thoroughly investigated and a review of the literature revealed that hot alkaline extraction is suitable for both WS and CS.

Alkaline pretreatment is among the most popular approaches for extracting hemicelluloses due to a series of advantages: highly efficient separation of hemicellulose, effective removal of acetyl groups from hemicellulose, mild reaction conditions, and relatively low operation costs [44,45,46,47]. The main drawback of alkaline pretreatments is the simultaneous removal of lignin that results in a lack of selectivity [47].

In this context, identifying the optimal processing parameters that permit the transition from one raw material (WS) to the other (CS), and vice versa, with minimal technological changes might be an important step towards real-life industrial applications and valorization of these particular LCB representatives. Several attempts to optimize the hot alkaline extraction of hemicelluloses from WS or CS have been reported so far which have all been based on a classic optimization method, i.e., the response surface methodology (RSM) [45,48,49,50,51,52,53,54,55].

To the best of our knowledge, this is the first documented attempt to investigate and optimize the technological possibilities of hemicellulose production using alternative dual feedstock corn stalks/wheat straw via hot alkaline extraction.

In this paper, we present an experimental study, including optimization methods, on hot alkaline extraction of hemicelluloses from WS and CS. The objectives are: (i) to establish the optimal conditions for the alkaline extraction of hemicelluloses from WS and CS using RSM; (ii) to separate the hemicelluloses from the resulting liquors; (iii) to perform the chemical characterization of the isolated hemicelluloses. Three process parameters were considered for optimization: time, temperature, and NaOH concentration (Table 1). The main hemicellulose components were xylan, arabinan, and glucan. The chemical characterization was performed using high performance chromatography (HPLC), Fourier transform infrared (FTIR) spectroscopy, proton nuclear magnetic resonance (1H NMR) spectroscopy, and thermogravimetric (TG) and derivative thermogravimetric (DTG) analyses.

## 2. Materials and Methods

### 2.1. Raw Materials Preparation and Analysis

The raw materials used in this study were wheat straw and corn stalks donated by local farmers in Iasi, Romania. The preliminary processing of the raw materials included: (i) removal of foreign materials, corn stalk leaves, dirt, and biodegraded parts; (ii) chopping to 10 cm pieces; (iii) grinding; (iv) sieving through a 1 mm sieve.

The following analytical methods were used to determine the quantities of the relevant chemical components of wheat straw and corn stalks (results reported in Table 2): (i) the Kurschner and Hoffer (ethanol-nitric acid) method for cellulose determination [56]; (ii) the Wise (sodium chlorite-acetic acid) method for holocellulose determination [57]; (iii) the TAPPI method of T 211 om-02 (2002) for ash [58]; (iv) the TAPPI method of T 207 om-88 for hot water extracts [59]; (v) the TAPPI method of T 212 om-02 (2002) for the 1% sodium hydroxide solubility [60]. Acid insoluble lignin (AIL) and acid soluble lignin (ASL) were determined using the NREL/TP-510-42618 method [61,62]. The known volumes of the G3 filtered hydrolysate in volumetric flasks were neutralized to a pH of 6 with barium carbonate (BaCO_3_). Aliquots of neutralized hydrolysate were used in the chromatographic analysis of the monosaccharide content. The used HPLC chromatography system (Agilent Infinity 1260) was equipped with a Phenomenex Rezex RPM-Monosaccharide Pb + 2 (8%) column, 300 × 7.8 mm, heated at 65 °C and a Shimadzu RID 10A refractive index detector (40 °C). The mobile phase consisted of ultrapure water with a flowrate of 0.6 mL∙min^−1^. The injection volume was fixed to 10 µL. Each sample and standard solution was filtered before injection by using 0.2 μm syringe PTFE Roth filters. Calibration curves in the concentration range of 0.05–0.3 g∙L^−1^ were constructed using solutions of 99% purity glucose, xylose, and arabinose (Flucka). The content of structural polysaccharides (glucan, xylan, and arabinan) in raw materials was determined using data on the monosaccharide concentrations of corresponding neutralized hydrolysate [61,62].

### 2.2. Experimental Design

Following similar studies in the literature [52,63,64,65,66,67], the main process parameters for extracting hemicelluloses from WS and CS were selected and optimized using response surface methodology (RSM). An initial screening set of experiments was performed to establish the variation ranges of the designated variables, which were reaction time, temperature, and alkali concentration, as shown in Table 1. The response variables were: Y1 and Y3 (the xylan extraction yield (XEY)) and Y2 and Y4 (the total extraction yield (TEY)) calculated using Equations (1) and (2), respectively. The experimental design and data processing were performed by using the Stat-Ease Design-Expert Software.

According to the RSM procedure, a minimum number of experiments (central composite design) were statistically programmed, as shown in Table 3 for WS and Table 4 for CS. The design included 15 experiments in total, as well as five replications at the center point. Then, the experimental results were used to generate equations describing the relationships between selected process parameters and model responses. Based on the dependences of total extraction yield and xylan extraction yield on the independent variables selected, the optimal conditions for the HAE of hemicelluloses were determined for WS and CS. For each raw material, quadratic models were proposed to correlate the response variable with the independent variables. Finally, after the optimal extraction conditions were determined, the model’s optimal conditions were experimentally validated. The liquors produced by these trials were saved and used to isolate hemicelluloses, which were later characterized.

### 2.3. Hot Alkaline Extraction of Hemicelluloses

The hot alkali extraction (HAE) procedure used to extract the hemicelluloses consisted of treating a weighted amount of 1 mm sieved material with sodium hydroxide solution (NaOH, Merck) in sealed reaction vessels at a solid to liquid ratio of 1:30 at different temperatures and for different periods of time, as shown in Table 3 and Table 4. Heating was ensured by means of a temperature-controlled ventilated oven. At the end of each experiment, the reaction vessels were chilled, and the contents of the sealed vessels were filtered on a G3 filtering crucible. The residual sediments in the filter were washed with sodium hydroxide solution at the same concentration as in the extraction experiment, followed by distilled water. The filtrate and washing liquids were collected in a volumetric flask and preserved for the homoxylan content analysis. Based on the initial homoxylan concentration of each type of raw biomass and the xylan content of the HAE liquor samples evaluated by HPLC, Equation (1) was used to compute xylan extraction yields.

(1) $$\mathrm{XEY}\;\left(\%\right)=\frac{m_{\mathrm{Xr}}-m_{\mathrm{XHAE}}}{m_{\mathrm{Xr}}}\cdot 100,$$

where XEY (%) represents the xylan extraction yield, m_XHAE_ is the absolute mass of the xylan determined by HPLC, and m_Xr_ is the absolute mass of the xylan in the raw material sample.

The xylose monomer in the extraction liquor samples was measured after the HAE liquors were treated with 4% sulfuric acid (60 min at 121 °C) according to the NREL (LAP) TP-510-42623 method [68]. The acid treatment completed the hydrolysis of the HAE dissolved polymeric carbohydrates. Following hydrolysis, the samples were neutralized and HPLC examined.

The solid residues on the filter were dried in a vacuum oven and then weighed. The weighing results were used to calculate the total extraction yield with Equation (2):

(2) $$\mathrm{TEY}\;\left(\%\right)=\frac{m_{o.d.}-m_{\mathrm{HAE}}}{m_{o.d.}}\cdot 100,$$

where TEY (%) represents the total extraction yield (the total amount of compounds extracted from the material); m_HAE_ is the mass of the solid residue remaining after the extractive treatment, oven dried; m_o.d._ is the mass of the acid treated samples, oven dried.

### 2.4. Separation and Characterization of Extracted Hemicelluloses

The ethanol precipitation method was used to separate hemicelluloses from the HAE liquors. In brief, 300 mL samples of alkali extraction liquors and soda pulping black liquor were mixed with 2 volumes of analytic purity ethanol (96%) and left to stand at 4 °C for 24 h. The precipitated hemicelluloses (HCs) were separated in three stages by centrifugation at a relative centrifugal force value of 2012 (3000 rpm) for a total time of 25 min in a Sorvall GLC2 equipped with an HL-4 rotor (100-mL bucket). The first stage (15 min) was used to separate the HC-containing solid precipitate, and the subsequent two centrifugation stages (each of 5 min) were used to separate the HC after ethanol washing. The acquired HC samples were dried at 50 °C and subjected to additional investigation.

The structural polysaccharide hemicelluloses composition was determined using the HPLC of the liquid obtained after the samples were hydrolyzed. To facilitate dissolution, from 60 to 80 mg were suspended in 10 mL of 0.1 M NaOH and vigorously shaken for at least 30 min. The complete hydrolysis was achieved by treating the samples with 4% sulfuric acid for 60 min at 121 °C. To determine the amounts of resulted monosaccharaides, neutralized samples of the obtained hydrolysate were analyzed by HPLC (Figures S1–S3, in the Supplementary Materials).

The color values (CVs) of precipitated hemicelluloses were determined by measuring the absorbance at 420 nm of the corresponding sodium hydroxide (2 wt.%) filtered solution samples (0.45 μm syringe PTFE Roth filter) [69]. A Jasco V550 UV-VIS spectrometer was used to record the absorbance values used in Equation (3):

(3) $$\mathrm{CV}=\frac{A_{420}}{b\cdot C},$$

where A_420_ denotes the value of absorbance at 420 nm, b is the optical path length, and C is the sugar concentration in g∙L^−1^.

The FTIR spectra of selected hemicellulose samples (WS and CS) were recorded using potassium bromide disks containing finely ground samples at 1% content on a Shimadzu IRAffinity-1S instrument (32 scans at 4 cm^−1^ resolution and 4000–400 cm^−1^).

The 1H-NMR spectroscopy data were obtained by dissolving amounts of 30 mg of hemicellulose samples in deuterated water, then pipetted into NMR tubes. Spectra were recorded on a Bruker Avance NEO 400 MHz spectrometer, operating at 400.1 MHz for 1H nuclei, with a 5 mm four nuclei direct detection z-gradient probe using standard pulse sequences, as delivered by Bruker with TopSpin 4.0.8 spectrometer control and processing software. The chemical shifts are reported in δ units (ppm), and were referenced to the sodium 3-(trimethylsilyl)-[2,2,3,3-d4]-1-propionate (TSP) internal standard at 0.0 ppm. For spectra registration, 128 scans were used.

The thermogravimetric analyses of the hemicellulose samples (WS and CS) were carried out using a Toledo TGA/SDTA 851 instrument at a heating rate of 10 °C∙min^−1^ and an air flow rate of 20 mL∙min^−1^. Ceramic pans were used to heat the samples from 25 °C to 900 °C. For data processing, the Mettler Stare SW 9.10 TGA/DTG software was used.

## 3. Results and Discussion

### 3.1. Chemical Composition of Raw Materials

Table 2 displays the chemical composition analysis results for the raw materials used in the current work. The relevant chemical components are cellulose, hemicelluloses, and lignin. Other important data in the table include the ash content, hot water extractives, and 1% sodium hydroxide extractives. While the content of hot water extractive gives an indication of the biomass’s soluble materials (tannins, gums, soluble sugars, starch, and coloring materials), the solubility of 1% NaOH is a much more complex indicator. The relatively dilute sodium hydroxide solution removes low molecular weight carbohydrates such as hemicelluloses and degraded cellulose, as well as some lignin. Environmental factors such as heat, moisture, oxygen, light, and microorganisms all play roles in the formation of these components. In some cases, such as cellulose production, 1% NaOH soluble may be considered to be a potential loss. The ash content is proportional to the amount of mineral substances present, and it is higher in non-wood biomass samples than in wood biomass samples. The experimental values obtained are consistent with those reported by other authors in similar studies [70,71].

The values reported in the literature differ greatly due to the variety of corn and the cultivation area. Variations in cellulose content may also occur as a result of the cellulose content determination methodology. The NREL method for cellulose determination involves hydrolysis to glucose in two steps of sample treatment: hydrolysis with sulfuric acid 72% and post-hydrolysis with sulfuric acid 4%. The results may be slightly higher than those obtained using gravimetric methods (i.e., ethanol-nitric acid). This is due to the quantification of cellulose as monosaccharides, which could result from cellulose hydrolysis, but also from hemicellulose branches [72].

### 3.2. The Influence of the HAE Process Parameters on Xylan and Total Extraction Yields

The experimental results of hot alkali extraction trials programmed using the central composite design are shown in Table 3 for WS and Table 4 for CS.

Second-order polynomial regression equations were used to fit the experimental data. The mathematical expressions were simplified by removing some non-significant terms while maintaining the model hierarchy. The proposed relationships among the xylan extraction yield, total extraction yield, and process parameters for WS HAE are shown in Equations (4) and (5).

(4) $$\mathrm{XEYws},\;\%=-237.70-0.54X_{1}+5.21X_{2}+16.44X_{3}+6.1910^{-3}X_{2}X_{3}+3.0710^{-3}X_{1}^{2}-0.03X_{2}^{2}-1.16X_{3}^{2},$$

(5) $$\mathrm{TEYws},\;\%=-56.17+0.22X_{1}+1.66X_{2}+4.51X_{3}-3.906\cdot 10^{-3}X_{1}X_{2}+6.9\cdot 10^{-4}X_{2}X_{3}+8.79\cdot 10^{-4}X_{1}^{2}-6.45\cdot 10^{-3}X_{2}^{2}-0.29X_{3}^{2},$$

Equations (6) and (7) show the expected relationships among the xylan extraction yield, total extraction yield, and process parameters for the CS HAE.

(6) $$\mathrm{XEYcs},\;\%=96.03-0.55X_{1}-0.567X_{2}+0.05X_{3}+3.86\cdot 10^{-3}X_{1}X_{2}+0.03X_{1}X_{3}+0.04X_{2}X_{3}-0.46X_{3}^{2},$$

(7) $$\mathrm{TEYcs},\;\%=-70.689-5.49\cdot 10^{-3}X_{1}+2.339X_{2}+0.06X_{3}+0.013X_{2}X_{3}-0.011X_{2}^{2},$$

The ANOVA analysis of the proposed model revealed that, in the case of wheat straw HAE, the F value is 50.46, indicating that the model is significant. The model’s *p*-value is less than 0.05, implying that the model terms are also significant. The values of the parameters such as adjusted R^2^ (0.94) and predicted R^2^ also indicate the model’s potential applicability in the design space, which is also confirmed by adequate precision, which is 19.34 and greater than 4.

Similar observation resides in the ANOVA analysis of the models proposed for corn stalk HAE. An F value of 37.71 is obtained in this case, indicating that the model terms are significant. The values of R’s also indicate the model’s adequacy, which is supported by a precision value greater than four.

The results of ANOVA analysis for both XEY and TEY are shown in Table 5, indicating that the proposed model is reasonably accurate.

### 3.3. Optimization of Hot Alkaline Extraction of Hemicelluloses from Wheat Straw

**Figure 1:**
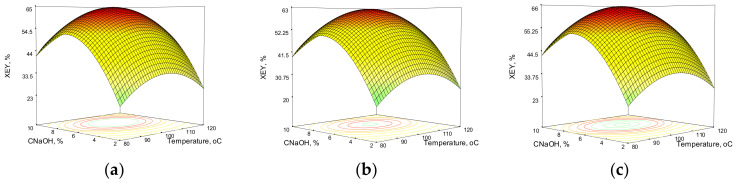
Response surfaces and contour plots of the combined effects of sodium hydroxide concentration and temperature on xylan extraction yield from wheat straw at various reaction times: (**a**) 60 min; (**b**) 90 min; (**c**) 120 min.

**Figure 2:**
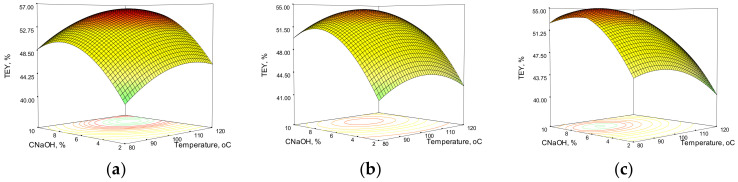
Response surfaces and contour plots of the combined effects of sodium hydroxide concentration and temperature on total extraction yield from wheat straw at various reaction times: (**a**) 60 min; (**b**) 90 min; (**c**) 120 min.

Figure 1 and Figure 2 show the response surfaces obtained from the model equations for xylan and total extraction yield in the case of wheat straw. These three-dimensional representations show the model responses as a function of sodium hydroxide concentration and temperature at various process times, emphasizing the effects of process variables on the results.

In the case of wheat straw HAE, the XEY does not increase with increasing C_NaOH_ across the entire temperature range. At concentrations higher than 7% NaOH in the extraction solution, the XEY begins to decline, most likely due to xylan macromolecular structure loosening and subsequent xylose breakdown in alkaline media.

At a constant sodium hydroxide charge, the influence of temperature could be noticed, right until 90–110 °C; the XEY continues to climb and, depending on the alkali concentration and process time, it also declines. The fluctuations caused by the temperature are not as severe as those induced by the alkali charge. However, the combined effect of temperature and alkali charge (interaction) must be considered because an increase in C_NaOH_ alone does not result in an increase in XEY at a constant temperature. Process time extension causes variations in the XEY, and extending the process time has a negative effect, but to a lesser extent than alkali concentration and temperature. The total extraction yield mostly follows the same trend as the xylan extraction yield, but it is worth noting that a high total extraction yield does not always correspond to a high value of xylan extraction yield, especially in higher severity conditions (high C_NaOH_, temperature, and process time). This could be due to the degradation of both the xylan chain and xylose in the liquor media during the HAE process, as previously mentioned. In addition to hemicelluloses, the HAE removes more than half of the lignin (52.4% from WS and 67.1% from CS), for a 72-min HAE with 7% wt. NaOH at 100 °C.

### 3.4. Optimization of Hot Alkaline Extraction of Hemicelluloses from Corn Stalks

**Figure 3:**
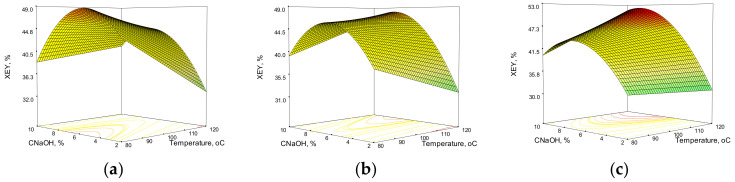
Response surfaces and contour plots of the combined effects of sodium hydroxide concentration and temperature on xylan extraction yield from corn stalks at various reaction times: (**a**) 60 min; (**b**) 90 min; (**c**) 120 min.

**Figure 4:**
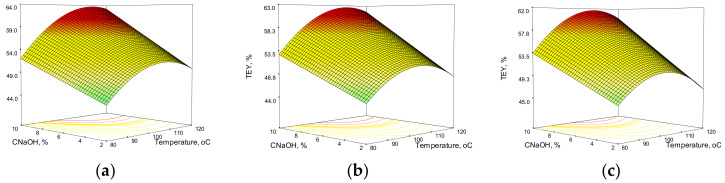
Response surfaces and contour plots of the combined effects of sodium hydroxide concentration and temperature on total extraction yield from corn stalks at various reaction times: (**a**) 60 min; (**b**) 90 min; (**c**) 120 min.

The response surfaces obtained from the model equations for xylan and total extraction yield in the case of corn stalks are shown in Figure 3 and Figure 4. The combined effects of temperature and C_NaOH_ on the XEY and TEY after 60, 90, and 120 min are highlighted in 3D representations.

Because the independent variables have different effects on the final process results, the response surfaces for the CS HAE and WS HAE differ. It is easy to notice that the values for xylan and total extraction yield are lower in the case of corn stalks than in the case of WS under similar conditions. C_NaOH_ has a strong influence on XEY, with a maximum value in the middle of the selected interval. In contrast to wheat straw HAE, where temperature influence is described by a second order polynomial, temperature influence on corn stalks is linear and negative. The effect of temperature on the XEY appears to be less pronounced as the HAE time increases. The total extraction yield also shows a strong dependence on alkali concentration, but this time it increased linearly. The parabola shape indicates a second order polynomial dependence in the case of temperature effect.

### 3.5. Optimal Values for the Wheat Straw and Corn Stalk HAEs

Maximum xylan extraction yield and maximum total yield were the optimization criteria for xylan extraction from wheat straw and corn stalks. Table 6 displays the values of the process parameters that ensure maximum yields, as well as the experimentally validated results. The standard deviation values are indicated in brackets. There are some acceptable differences between the predicted and experimental values of XEY and TEY. The differences are smaller in the case of TEY, indicating that the obtained model behaved better in the so-called design space.

As expected, the nature of the raw materials causes some variation in the optimal values of the main process parameters of the HAE. In the case of a production line that uses wheat straw and corn stalks as raw materials in succession, the periodicity of the seasonal transition from WS to CS necessitates some reasonable changes in order to maximize yields. According to the data in Table 6, an increase in temperature of 16 °C, an increase in alkali concentration from 7 to 9 wt.%, and an additional 46 minutes of batch time are not technologically insurmountable.

Another feasible option is to keep the process parameters unchanged during and after the seasonal transition from WS to CS. Less energy consumption, a smaller amount of chemicals, and a consistent reduction in the batch time might justify a drop of 12% of the xylan extraction yield accompanied with a minor increase in the total extraction yield. According to this point of view, the hemicellulose characterization was carried out on the samples obtained under identical raw material treatment conditions. The optimal conditions obtained and validated for the HAE of wheat straw were chosen for this purpose because they are moderate and require fewer chemicals, energy, and processing time.

The following discussion is included concerning the reliability of the hemicellulose extraction yields from wheat straw and corn stalks. The variation in hemicellulose extraction yields is caused by two factors: (i) natural factors (including WS and/or CS assortments and their seasonal periodicity that affects the raw materials quality in terms of humidity, lignin content, and so on) and (ii) technological factors (including the types of pretreatments and the extraction’s process parameters (Table 7), such as temperature, NaOH concentration, and time). The former category is rather unpredictable, whereas the latter category can be controlled, optimized, and reproduced. The “natural” yield alteration can be appraised in the range of ±10% while the “technical” yield variation can fluctuate significantly, as presented in Table 7.

Taking all of these factors into account, determining the optimal process parameters that will allow the transition from one raw material (WS) to the other (CS) with minimal technological changes is critical as a first step in scaling up from the laboratory to pilot and industrial levels. However, a few compromises are required, and some simplifications (e.g., reducing the number of technological parameters) would facilitate the use of complementary raw materials such as wheat straw and corn stalks, despite reductions in the extraction yields.

### 3.6. Hemicellulose Characterization: Wheat Straw vs. Corn Stalks

The HPLC analysis of dried specimens obtained by ethanol separation of the HAE liquors obtained under identical treatment conditions revealed the chemical composition of the separated hemicellulose samples (Table 8). The main macromolecular component in both samples is xylan. The results for arabinan content support the structure of arabinoxylan-type hemicelluloses, as described by other authors for cereal-derived hemicelluloses [75,79]. The contents of the main chemical components discovered in this study are similar to those reported in other studies [69,80]. The CS-isolated sample appears to have about 5% more xylan than the WS hemicellulose preparations in terms of xylan content as the main polysaccharide [80].

**Figure 5:**
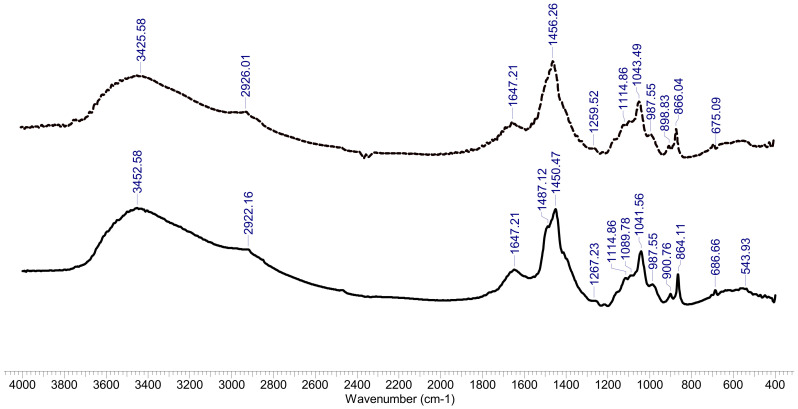
FTIR spectra of separated hemicelluloses. Black line, HCWS and dotted line, HCCS.

The bands in the FTIR spectra of the hemicellulose samples (Figure 5) were assigned as follows: the band occurring at ~3400 cm^−1^ was assigned to the stretching vibrations of the O-H groups; the band occurring at ~2950 cm^−1^ was assigned to the -CH_2_ antisymmetric stretching, while the band at 2850 cm^−1^ was a result of -CH_2_ symmetric stretching; the band occurring at ~1650 cm^−1^ was assigned to the absorbed water [81]. The strong band occurring at about 1450 cm^−1^ could be assigned to the presence of the methyl groups. Peaks are visible at ~1100 and ~1040 cm^−1^ of C-O stretching in the C-O-C ether linkages (the first is the inter sugar units and the second results from intra sugar (in alcoholic functional group). The peaks at ~895 cm^−1^ were attributed to the stretching vibration modes (both symmetric and antisymmetric) of C-O in the -C ether linkage. In fact, the 895 cm^−1^ peak is specific to the β-1-4 bonds between xylose units of the xylan chain [82]. Other bands at lower wavenumbers, such as ~690 cm^−1^, could be attributed to the out-of-plane C-H deformations.

**Figure 6:**
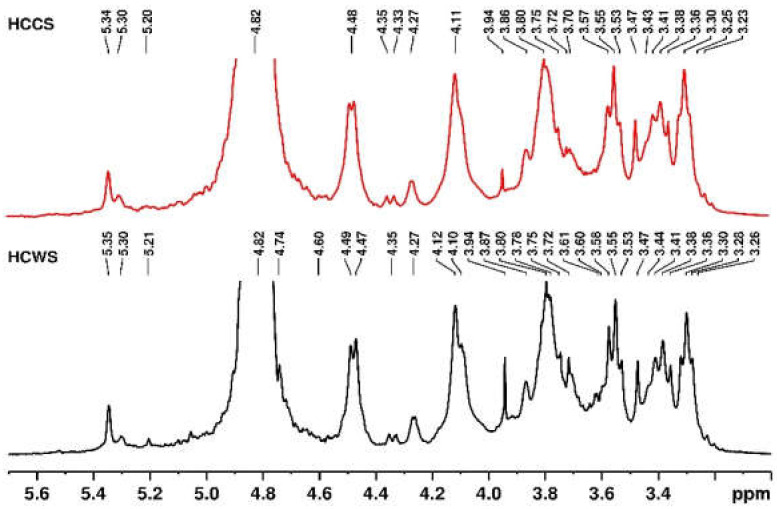
1H-NMR spectra of separated hemicelluloses. Black line, HCWS and red line, HCCS.

In the 1H-NMR spectra displayed in Figure 6, several signals can be observed and analyzed. According to the literature [83], spectral data could be classified in two regions: the β-(1-4)-D-anhidroxylopyranose units heterocycle proton region (4.4–3.0 ppm) and the anomeric regions, namely 5.5–4.9 for α-anomers and 4.9–4.4 for β-anomers [84]. In Figure 6 (HCWS spectra) there are five different anomeric groups, four signals are assigned to β units (reported between 4.9 and 4.4 ppm) and two signals are attributed to α units (reported between 5.3 and 5 ppm). Therefore, the signals are attributed to β-xylose (X), glucuronic acid substituted β-xylose (XG), β-glucose (G), and α-glucuronic acid (AG) (Figure S4 and Table S1 in Supplementary Materials). All structures for these units have been reported in the literature [85]. The observable signals for α-L-arafuranosyde residues (Ara) are assigned to the anomeric proton H1_Ara_ at 5.35 ppm and for H4_Ara_ proton (see Figure S4 in Supplementary Materials) at 4.27 ppm [86]; the remaining signals overlap with other signals in the spectrum. Thus, for the superposed peaks, the attribution was made according to the data previously reported [83]: H2_Ara_, 4.08 ppm; H3_Ara_, 3.76 ppm; H5_Ara_, 3.73 ppm. The signals in the interval 4.49–4.47 (peak at 4.48 ppm) are due to the anomeric proton H1_X_ of β-D-xylopyranoside residues (or β-xylose, noted above with X) [85,87,88], and the remaining signals are assigned as follow: 4.12 ppm, H5eq _X_; 3.80 ppm, H4_X_; 3.55 ppm, H3_X_; 3.38 ppm, H5axx3.30 ppm, H2_X_.

**Figure 7:**
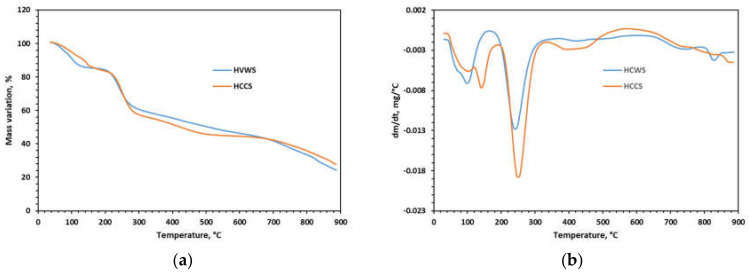
(**a**) Mass variation curves; (**b**) DTG curves, for the separated hemicellulose samples.

A thermogravimetric analysis is an important tool for studying the thermal stability of materials. The mass variation and the DTG curves obtained for the hemicellulose samples are displayed in Figure 7a,b. In general, the mechanism of the thermal decomposition of polymeric materials involves three main phases: water loss (drying), macromolecular chain degradations (depolymerization and pyrolysis), and finally char oxidation. The plots of the HCWS and HCCS samples are relatively similar, but some differences occur. In the case of the mass variation plot of HCWS, at least three main stages were observed: the first stage, between 52 °C and 125 °C, corresponds to complete dehydration (mass loss of 15.29%), the second stage corresponds to degradation, starting at 216 °C and ending up at 275 °C, with a mass loss of 28.9%; and the last stage, starting at about 360 °C and ending at 470 °C, is char oxidation. Peak temperature values (71 °C, 220 °C, and 420 °C) for the thermal decomposition of the HCWS are observable from the DTG curves (Figure 7b), which show the temperatures with the highest rates of reaction.

The HCCS behavior under heating is a little different. In this case, at least four zones in the TG curves were identified. The first zone corresponds to drying (52–132 °C, peak at 89.9 °C, 10.58% mass loss), followed by a second zone probably caused by a loss of some volatiles (132–224 °C, peak at 142 °C, 13.5% mass loss). The third zone is the thermal decomposition of the polysaccharide and is observed between 224 and 366 °C with a peak rate at 245 °C and 21.05% mass loss. A final zone corresponding to char oxidation can be clearly observed between 366 °C and 489 °C. For both of the samples, the final parts of the plots address the chemical transformations of the mineral part of the material. Overall, the hemicellulose samples show similar thermal stability, which corresponds to xylan-based polysaccharides [89].

## 4. Conclusions

The response surface methodology proved to be a valuable tool for optimizing the hot alkaline extraction process used to extract hemicelluloses from wheat straw and corn stalks. For the wheat straw, the optimal values for the parameters considered are 72 min, 7% sodium hydroxide concentration, and a temperature of 100 °C, whereas the values are different for the corn stalks, i.e., 118 min, 9% sodium hydroxide concentration, and 120 °C. In both cases, xylan was found to be the most abundant chemical component, followed by arabinan and glucan. Under the same process parameters, the raw materials performed differently, yielding different amounts of extracted xylan: 87.83% for WS and 92.95% for CS. As for the arabinan, the wheat straw produced almost a double amount (11.09%) as compared with the corn stalks (6.44%). The amount of glucan was very small, yet higher for WS (1.08%) than for CS (0.64%).

The chemical characterization of the hemicellulose samples obtained under optimal conditions proved that they belong to the class of arabinoxylans, a type of polysaccharides specific to grass and cereal plants. The FTIR and 1H-NMR data, as well as the results of the thermogravimetric study, confirmed the presence of xylan as the main component.

WS and CS are both suitable as raw materials for hemicellulose production (xylan, arabinan, and glucan) and hot alkaline extraction is the appropriate pretreatment method. The seasonal transition from WS to CS can be solved technologically by: (i) making moderate changes to the main process parameters to ensure high extraction yields for both WS and CS and (ii) keeping the parameters constant (WS) to reduce energy, chemicals, and time consumption, resulting in a decrease in XEY for the CS. The study’s significant impact is the ease of transition from laboratory experimental setup to industrial facility with potential integration into other processes resulting in biomass value addition. Some economic studies are required at this point to support an appropriate technological decision.

## Figures and Tables

**Table 1:** Independent variables and variation range for the HAE of hemicelluloses from WS and CS.

Independent Variables	Measure Units	Code	Range		Symbol
			from	to	
Reaction time	minutes	X1	60	120	t
Temperature	°C	X2	80	120	T
NaOH concentration	wt.%	X3	2	10	C_NaOH_

**Table 2:** Chemical compositions of WS and CS used as raw materials for the HAE of hemicelluloses.

	C (%)	HC (%)		L (%)		1% NaOH (%)	HWE (%)	Ash
WS	42.9 (0.40) ^1^ 40.87 (0.98) ^2^	35.45 ^3^ (0.77)		20.25 ^4^		44.36 (0.66)	11.85 (0.18)	4.55 (0.25)
		X^2^, %	22.5 (0.72)	AIL, %	18.5 (0.65)			
		A^2^, %	5.34 (0.65)	ASL, %	1.75 (0.12)			
CS	45.09 (0.87) ^1^ 43.68 (1.19) ^2^	27.35 ^3^ (1.31)		23.58 ^5^		43.09 (0.87)	15.44 (0.18)	6.53 (0.36)
		X^2^, %	19.64 (1.14)	AIL, %	22.12 (0.89)			
		A^2^, %	3.42 (0.89)	ASL, %	1.46 (0.28)			

WS—wheat straw; CS—corn stalks; C—cellulose; HC—hemicelluloses; A—arabinan; X—xylan; L—lignin; ^1^ determined by ethanol-nitric acid method; ^2^ determined by NREL method and subsequent HPLC analysis; ^3^ determined as the difference between holocellulose (sodium chlorite method) and cellulose content (ethanol-nitric acid method); ^4^ determined as the sum of acid insoluble lignin (AIL) and acid soluble lignin (ASL) according to the NREL method; ^5^ hot water extractives determined by the TAPPI method of T 207 om-88. Data are presented as means of triplicates and values in the brackets correspond to standard deviations determined.

**Table 3:** Xylan and total extraction yield results for WS HAE.

Exp.	Time (minutes)	Temperature (°C)	C_NaOH_ (%)	XEY_WS_ (%)	TEY_WS_ (%)
	X_1_	X_2_	X_3_	Y_1_	Y_2_
1	60	80	2	23.45	40.70
2	120	80	2	19.44	44.20
3	60	100	2	37.23	44.52
4	120	100	2	41.51	46.85
5	60	120	2	20.78	45.80
6	120	120	2	28.57	40.40
7	60	100	6	61.21	53.27
8	90	100	6	61.73	54.20
9	90	100	6	60.98	54.10
10	90	100	6	62.04	52.95
11	90	100	6	61.25	53.50
12	90	100	6	60.88	53.17
13	90	100	6	61.05	53.83
14	60	80	10	47.08	49.65
15	120	80	10	41.05	51.80
16	60	100	10	52.33	54.95
17	90	100	10	49.96	51.44
18	120	100	10	51.32	53.20
19	60	120	10	48.34	55.40
20	120	120	10	50.21	47.70

**Table 4:** Xylan and total extraction yield results for CS HAE.

Exp.	Time (minutes)	Temperature (°C)	C_NaOH_ (%)	XEY_CS_ (%)	TEY_CS_ (%)
	X_1_	X_2_	X_3_	Y_1_	Y_2_
1	60	80	2	44.36	44.09
2	120	80	2	30.88	45.80
3	90	100	2	34.59	51.03
4	60	120	2	32.34	50.36
5	120	120	2	32.37	45.78
6	90	80	6	35.48	48.03
7	60	100	6	45.90	57.70
8	90	100	6	50.15	54.33
9	90	100	6	45.20	57.50
10	90	100	6	47.60	54.45
11	90	100	6	46.21	53.05
12	90	100	6	47.80	54.50
13	90	100	6	46.50	57.10
14	120	100	6	46.20	58.90
15	90	120	6	43.85	56.55
16	60	80	10	37.23	53.19
17	120	80	10	40.58	53.24
18	90	100	10	42.67	59.90
19	60	120	10	42.40	56.45
20	120	120	10	50.78	60.55

**Table 5:** ANOVA analysis of the model’s parameters.

Raw Material	Model Response	F Value	*p*-Value	Adjusted R^2^	Predicted R^2^	Adequate
WS	Y_1_	50.46	<0.0001	0.9398	0.8746	19.34
	Y_2_	47.34	<0.0001	0.9447	0.8584	19.93
CS	Y_3_	37.71	<0.0001	0.9379	0.8175	20.51
	Y_4_	23.56	<0.0001	0.8758	0.7270	15.79

**Table 6:** The comparison between the model predicted and optimal experimental values.

Raw Material	Time (minutes)	Temperature (°C)	C_NaOH_ (%)	Predicted XEY (%)	Predicted TEY (%)	Experimental XEY (%)	Experimental TEY (%)
WS *	72	100	7	62.65	54.68	61.82 (1.57)	51.60 (2.45)
CS	118	116	9	50.85	57.03	52.91 (0.89)	60.62 (1.24)
CS *	72	100	7	47.26	55.87	46.89 (1.74)	53.90 (1.89)

* HAE liquors chosen for further separation of hemicelluloses as described in the Methods section.

**Table 7:** Hemicellulose extraction yields from wheat straw and corn stalks.

Raw Material	HC Yield	Process Conditions	Ref.
**Wheat straw**	83%	1.5% NaOH (*w*/*v*); 20 °C; 144 h multiple stage and multiple hemicellulose fractions; preliminary chlorite/acetic acid removal of lignin	[73]
	81%	10% NaOH (*w*/*v*); S/L ratio of 1:40; 20 °C; 6 h	[73]
	56.1%	10% NaOH (*w*/*v*); S/L ratio of 1:20; 40 °C; 90 min	[74]
	33.3%	10% NaOH (*w*/*v*); S/L ratio of 1:14; 40 °C; 90 min	[75]
**Corn stalks**	72%	4% NaOH (*w*/*v*); S/L ratio of 1:100; 30 °C; 18 h	[76]
	65%	10% NaOH (*w*/*v*); S/L ratio of 1:20; 20 °C; 10 h; (pre-treatment with hot water; S/L ratio of 1:20; 80 °C; 2 h; and lignin removal by chlorine dioxide treatment)	[77]
	80%	10% NaOH (*w*/*v*); S/L ratio of 1:10; 90 °C; 2 h	[78]

S/L, solid to liquid ratio; *w*/*v*, weight/volume concentration.

**Table 8:** Main chemical components of the hemicellulose fractions isolated by HAE.

Sample	Glucan (%)	Xylan (%)	Arabinan (%)	Purity (%)	Color Value 10^4^
HCWS	1.08	87.83	11.09	66.9%	9.74
HCCS	0.56	92.95	6.44	74.2%	8.75

## Data Availability

The data supporting the reported results is presented in the manuscript.

## Supplementary Materials

The following supporting information can be downloaded at https://www.mdpi.com/article/10.3390/polym14091662/s1: 1H-NMR spectroscopy data and HPLC chromatograms for the separated hemicelluloses. Figure S1: HPLC chromatogram of neutralized HCWS hemicellulose acid hydrolysate, Figure S2: HPLC chromatogram of neutralized HCCS hemicellulose acid hydrolysate, Figure S3: HPLC chromatogram of monosaccharide mixture containing cellobiose, Figure S4: ^1^H NMR spectrum of HCWS sample with signals assignment, recorded in D_2_O, and the labeled chemical structural units, Table S1: ^1^H NMR Signals assignment of hemicelluloses samples extracted from HCWS and HCCS in D_2_O.
