# Supplementary material for: An Experimental Study on the Hot Alkali Extraction of Xylan-Based Hemicelluloses from Wheat Straw and Corn Stalks and Optimization Methods
Source: Polymers (Basel). 2022 Apr 20;14(9):1662. doi: 10.3390/polym14091662 (PMC9102963; doi:10.3390/polym14091662)
Supplement: Supplementary file 1 [file polymers-14-01662-s001.zip › polymers-1654962-supplementary.pdf]

Supplementary material, 2nd revision

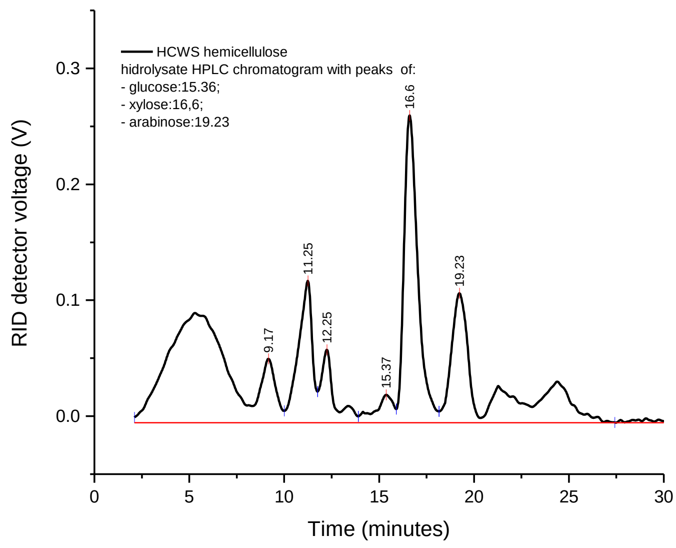

Figure S1 HPLC chromatogram of neutralized HCWS hemicellulose acid hydrolysate

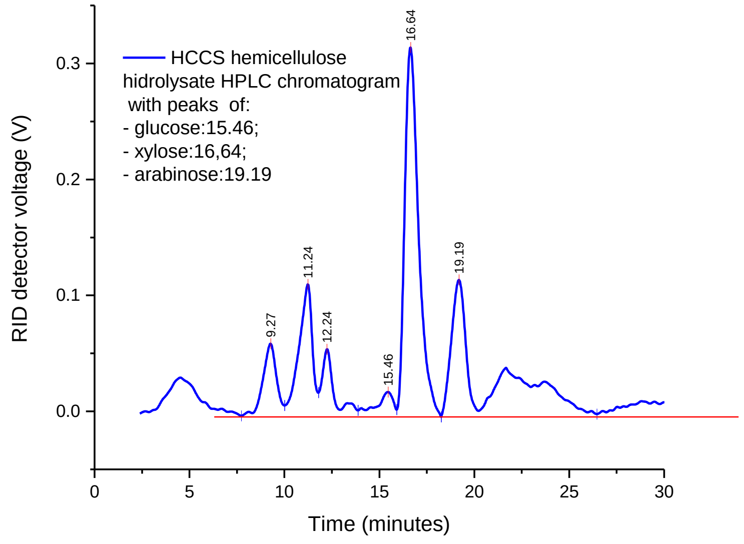

Figure S2 HPLC chromatogram of neutralized HCCS hemicellulose acid hydrolysate

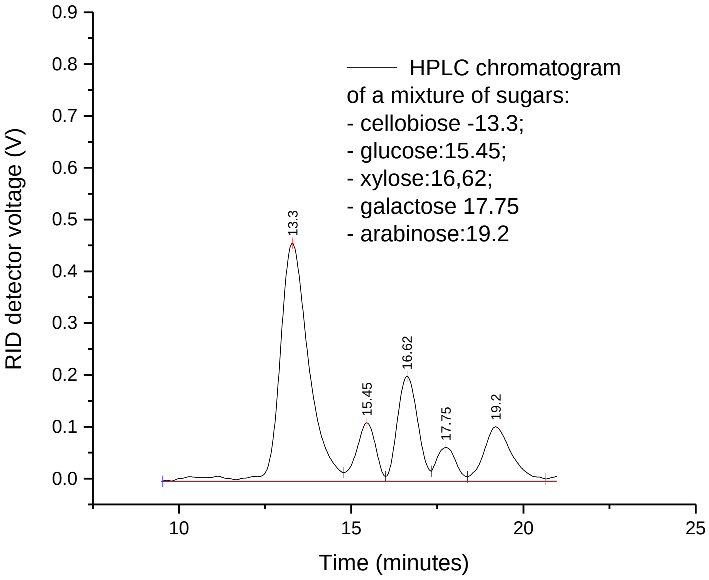

Figure S3 HPLC chromatogram of monosaccharide mixture containing cellobiose (0.063g·L^{-1}), glucose (0.024g·L^{-1}) , xylose (0.052g·L^{-1}), galactose (0.045g·L^{-1}) and arabinose (0.023g·L^{-1}) –injection volume 25uL

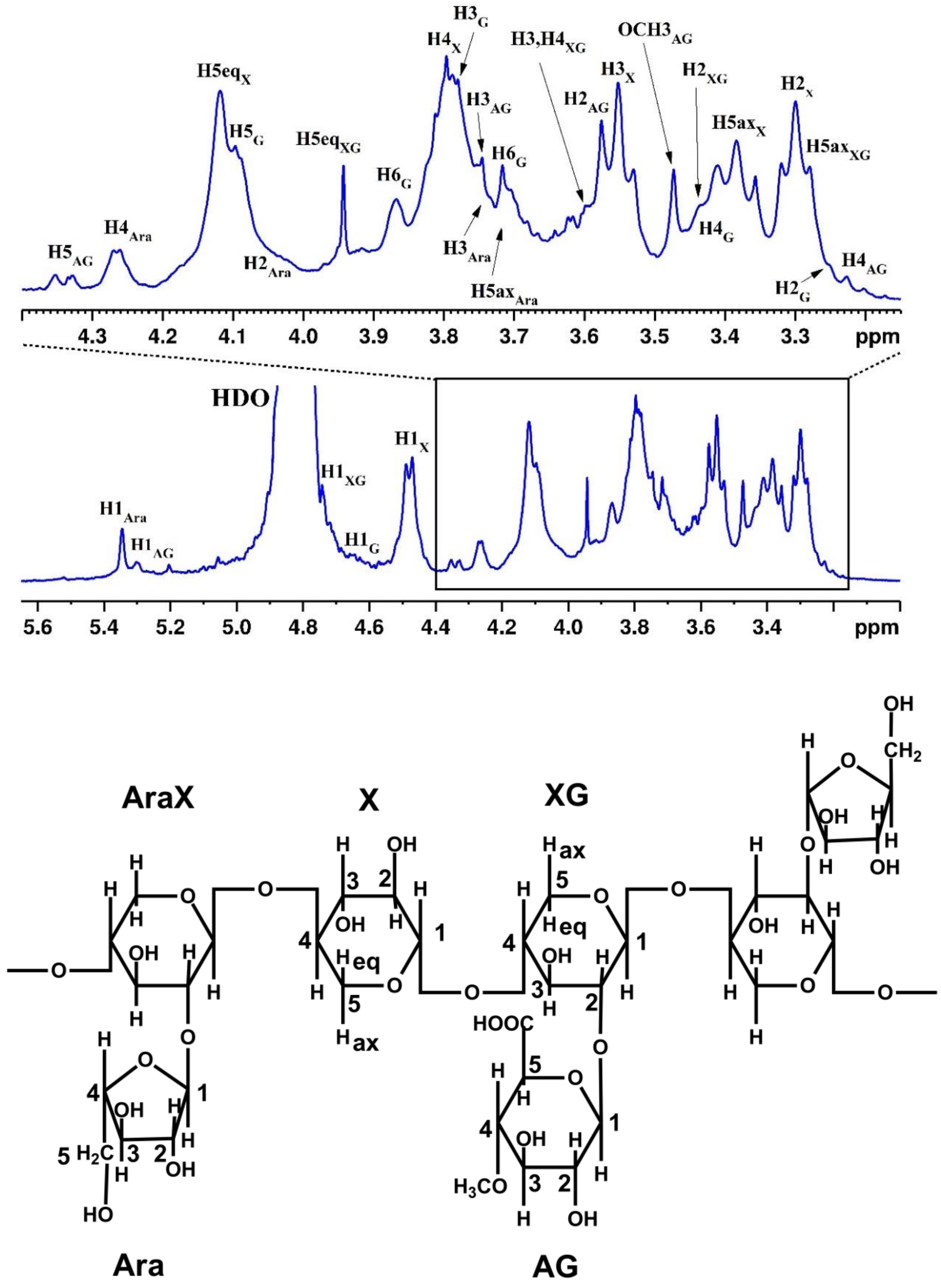

Figure S4: ^{1}H NMR spectrum of HCWS sample with signals assignment, recorded in D_2O, and the labeled chemical structural units.

Table S1: ^{1}H NMR Signals assignment of hemicelluloses samples extracted from HCWS and HCCS in D_2O

Hemicelluloses	Monosaccharide units	Position	δ ^{1}H (ppm)
Methylglucuronoxylans	β-Xylose non-substituted (**X**)	1	4.48
		2	3.30
		3	3.55
		4	3.80
		5_{ax}	3.38
		5_{eq}	4.12
	β-Xylose substituted (**XG**)	1	4.74
		2	3.43
		3	3.60
		4	3.60
		5_{ax}	3.28
		5_{eq}	3.94
	4-O-Methyl-α-Glucuronic acid (**AG**)	1	5.30
		2	3.58
		3	3.75
		4	3.23
		5	4.34
		-COOH	-
		-OCH3	3.47
	β-Glucose (**G**)	1	4.60
		2	3.26
		3	3.78
		4	3.43
		5	4.10
		6	3.72 and 3.87
